# Preconcentration of Superbase Ionic Liquid from Aqueous Solution by Membrane Filtration

**DOI:** 10.1021/acs.iecr.2c02217

**Published:** 2022-09-23

**Authors:** Filipe H. B. Sosa, Pedro J. Carvalho, João A. P. Coutinho

**Affiliations:** CICECO − Aveiro Institute of Materials, Department of Chemistry, University of Aveiro, Aveiro 3810-193, Portugal

## Abstract

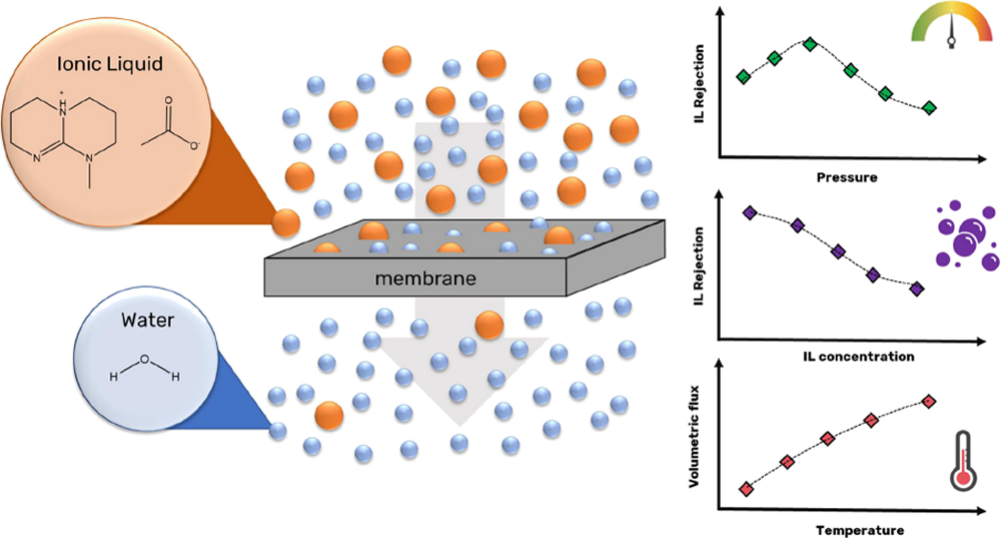

Certain organic superbase ionic liquids (ILs) have shown good cellulose dissolution and fiber regeneration performance, allowing us to obtain high-quality textile fibers. However, there is a lack regarding the IL recovery from the spinning bath and its purification, which is essential for the economic viability of the process. Aiming to understand methods to separate ILs from water for reuse/recycle, the use of pressure-driven membrane processes to recycle ionic liquids from aqueous solution was investigated. The recovery of two superbase ILs, 7-methyl-1,5,7-triazabicyclo[4.4.0]dec-5-enium acetate, [mTBDH][OAc], and 5-methyl-1,5,7-triaza-bicyclo[4.3.0]non-6-enium acetate, [mTBNH][OAc], were studied using different types of membranes (microfiltration, ultrafiltration, nanofiltration, and reverse osmosis, RO). Additionally, pressure, IL concentration, temperature, and multicycle effect were evaluated. Significant retentions (>45%) were obtained for the nanofiltration and RO membranes (NF270-NF and BW30LE-RO). The increase in pressure and temperature resulted in an increase in volumetric flux and a decrease in IL retention. On the other hand, IL concentration decreased the volumetric flow and rejection. For the serial filtration tests, a three-fold ionic liquid concentration was achieved, for a maximum concentration of 14 wt % of the ionic liquid. The membrane filtration methodology proved to be an efficient technique for carrying out the preconcentration of the IL from dilute solutions.

## 1 Introduction

The global textile fiber production has almost doubled in the last 20 years, from 58 million tons in 2000 to 109 million tons in 2020. It is estimated that the demand for textile fibers will continue to increase, with a projected production of 134 million tons by 2030, because of population growth and increasing personal consumption.^1^ The textile fiber market mainly comprises synthetic fibers (about 60%), cotton fibers (30%), and the remaining cellulosic fibers and other natural fibers.^2^ Synthetic fibers are mainly produced with nonrenewable resources and depend on declining fossil oil resources. On the other hand, wood-based fibers are generally produced from cellulose pulps. However, cellulose must be first dissolved to be used to produce fibers.^3^ In industry, the widely used Viscose process (carbon disulfide) produces a large amount of residues (alkaline and acid residues, toxic gases), and in the Lyocell process the explosive NMMO (*N*-methylmorpholine *N*-oxide) can cause environmental problems.^4^ Therefore, there is a clear need for an alternative sustainable solvent system to produce artificial cellulose fibers. Recently, ionic liquids (ILs) have been proposed as sustainable alternative solvents to produce these fibers.^5,6^ Of the several ILs identified as capable of dissolving cellulose, only a small fraction has the characteristics suitable to produce regenerated cellulose fiber (excellent cellulose dissolution and acceptable spinning properties).^7^ Recently, 7-methyl-1,5,7-triazabicyclo[4.4.0]dec-5-enium acetate, [mTBDH][OAc], and 5-methyl-1,5,7-triaza-bicyclo[4.3.0]non-6-enium acetate, [mTBNH][OAc], have been identified as promising solvents to produce high-performance fibers.^8,9^ Elsayed et al.^10^ observed in a dissolution study a superior tolerance of [mTBDH][OAc] to solvent-induced changes (water, hydrolysis products and A/B ratio) when compared to [DBNH][OAc]. In this study, good cellulose dissolution was achieved even with a high water content of 10 wt % (58 mol %), demonstrating a pronounced tolerance of [mTBDH][OAc] to the presence of water. Furthermore, [mTBDH][OAc] and [mTBNH][OAc] was more hydrothermally stable than [DBNH][OAc], and the same was found for the stability of the fiber spinning process. In general, these superbases IL have a high potential to be applied in the Ioncell process.

However, after fiber regeneration, the spinning bath contains a variety of contaminants, such as IL, water, and fragments from unregenerated cellulose and some degradation products.^11,12^ The recovery of the ILs and their purification is crucial from both an environmental and an economic perspective (Figure S1).

For the separation of water from an IL, evaporation is widely used because of the low vapor pressure of ILs.^13^ However, this process consumes a lot of energy and requires high temperatures, for which some ILs can be degraded. Furthermore, the IL low volatility can become a problem separating low-volatile solutes (carbohydrates, salts) and heat-sensitive products.^14^ Among the various processes used for separation/recovery of ILs, it is possible to highlight adsorption (activated carbon, resin),^15^ extraction (organic solvents, scCO_2_),^16^ crystallization,^17^ force field,^18^ distillation,^9^ and membranes.^19,20^

Membrane separation processes are widely used in the industry as they are cost-effective, involve simple operation, and exhibit high efficiency. This methodology is widely used for the treatment of water and sewage.^22^ The study with commercial membranes allows the rapid scale-up of the process because these membranes are available on a large scale on the market. In addition, the variety of commercial membranes available enables the selection of the most suitable for each process.

In this sense, the application of membranes (nanofiltration, reverse osmosis (RO), and pervaporation) was investigated to purify ILs.^13,21,22^

Along with membrane-based techniques, nanofiltration is one of the most studied techniques to concentrate ILs because of the high purity of the permeates and economical operation. Kröckel and Kragl^19^ were one of the first authors to report the application of nanofiltration membranes to separate [C_4_C_1_im][BF_4_] and bromophenol blue in aqueous solution and [C_1_C_1_im][CH_3_SO_4_] from lactose. Bromophenol blue and lactose were retained on the membrane while the IL permeated the membrane. Han et al.^23^ used nanofiltration to recover ionic liquids from reactions mediated by ILs. The authors report a rejection efficiency of almost 95% for ICYPHOS101 and ECOENG500 ILs in methanol and ethyl acetate solutions using STARMEM 120 and 122 nanofiltration membranes. In another study, Hazarika et al.^24^ studied the effect of lignocellulose concentration and applied pressure gradients on IL rejection with a commercial nanofiltration membrane (NF270-400, FilmTech). More than 50% of IL was retained by the membrane, with the solvent flow able to be manipulated and increased by increasing the retentate pressure. Comparably, Wang et al.^25^ observed that the permeate flux increases with applied pressure when recovering [C_4_C_1_im]Cl (a recovery rate of up to 96%) with a commercial nanofiltration membrane (NF90-DOW-Filmtec). Abels et al.^26^ showed that higher IL concentrations led to a decrease in permeate flux because of low IL permeability and osmotic pressure differences. Haerens et al.^27^ reported that the osmotic pressure was the limiting factor on the IL/water separation, for nanofiltration and RO membranes. The authors describe only an achievable five-fold ionic liquid concentration for a maximum concentration of 20–25 vol % of the IL. Therefore, membrane processes can hardly be used as a single step for separating IL from water because the osmotic pressure of the target concentration (1–3 wt % of water) would exceed the technical possibilities, so another methodology separator must be used together.

From this perspective, the objective of this work was to preconcentrate the IL from a synthetic spinning bath solution using membranes. Therefore, the performance of two superbase-based IL, 7-methyl-1,5,7-triazabicyclo[4.4.0]dec-5-enium acetate, [mTBDH][OAc], and 5-methyl-1,5,7-triaza-bicyclo[4.3.0]non-6-enium acetate, [mTBNH][OAc], that are good candidates to produce high performance cellulose fibers, was studied under different operation conditions. The IL retention, volumetric flux, pressure effect, IL concentration, and temperature were evaluated. In addition, the multicycle series of nanofiltration and RO membranes were evaluated for the purification of IL from an aqueous solution.

## 2 Experimental Section

### 2.1 Chemicals

The superbase-based ILs 7-methyl-1,5,7-triazabicyclo[4.4.0]dec-5-enium acetate, [mTBDH][OAc] (purity >99%), and 5-methyl-1,5,7-triaza-bicyclo[4.3.0]non-6-enium acetate, [mTBNH][OAc] (purity >97%) were synthesized at the University of Helsinki by a stoichiometric mixture (1:1) of acetic acid and the respective superbase (mTBDH or mTBNH), as described elsewhere.^28^ In summary, the base was placed in a bottom flask and stirred with a magnetic bar, while acetic acid was added dropwise to the base at 80 °C to avoid crystallization. The purity and structure of ILs synthesis were checked by ^1^H-NMR (Figures S2 and S3). The water content of the ILs was determined through the use of a Metrohm 831 Karl-Fischer coulometer, with the analyte Hydranal-Coulomat AG from Riedel-de Haën. The ultrapure water used to prepare the aqueous solution of ILs was double-distilled, passed through an RO system, and further treated with a Milli-Q plus 185 water purification apparatus.

### 2.2 Filtration Procedure

Filtration experiments have been carried out in a stirred cell for flat sheet membranes (Sterlitech HP4750; *V*_max_: 300 mL, *p*_max_: 69 bars, active membrane area 14.6 cm^2^). First, the cell was filled with 75 mL of IL solution, sealed, and then pressure (nitrogen, 10–50 bars) was applied to permeate the solution. The permeate collected represents a decrease in volume of the original feed solution of 5 to 25%. Supplementary experiments indicate that pseudo steady-state operation is attained until about 25% of the original feed volume is permeated (Figure S5). All membranes (MP005-MF, PT-UF, GH-UF, DL-NF, TS80-NF, NF270-NF, BW30LE-NF, UTC-73A-RO) were flushed with pure water before the experiments. The solution to be permeated was constantly stirred at 200 rpm (SCILOGEX SCI550-Pro, hotplate stirrer) to ensure the homogeneity of the system. The permeate was collected in a beaker under an analytical balance (Sartorius LA2000P, *d* ± 0.001 g) and was quantified over time. The permeate volume was collected for 30 to 60 min, and this value was used to calculate the volumetric flux (eq 1).
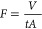
1where *F* is the volumetric flux, *V* is the volume collected in time *t* and *A* is the membrane area.

Initial membrane screening was performed with microfiltration, ultrafiltration, nanofiltration, and RO membranes. The detail characteristics of membranes are presented in the supplementary material (Table S1).

Studies with the RO membrane (BW30LE-RO) and nanofiltration membrane (NF270-NF) were conducted at five pressures (10, 20, 30, 40, and 50 bars) with a controlled temperature of 298.2 and 313.2 K and different feed concentrations (Table 1). The BW30LE-RO and NF270-NF membranes were chosen because they were designed to operate at lower pressures, with similar fluxes.^27^

**Table 1:** List of Membranes, Feed Streams, and Tested Conditions

membrane	conditions (agitation 200 rpm)		
	IL concentration	pressure	temperature
MP005-MF, PT-UF, GH-UF, DL-NF, TS80-NF, NF270-NF, BW30LE-NF, UTC-73A-RO	1 wt % [mTBDH][OAc]	10 bars	298.2 K
	1 wt % [mTBNH][OAc]	10 bars	298.2 K
BW30LE-RO, NF270-NF	1 wt % [mTBDH][OAc]	10–50 bar	298.2 K
	1 wt % [mTBNH][OAc]	10–50 bar	298.2 K
	1, 5, 10, 15, 20 wt % [mTBDH][OAc]	40 bars	298.2 K
	1, 5, 10, 15, 20 wt % [mTBNH][OAc]	40 bars	298.2 K
	1, 15 wt % [mTBDH][OAc]	40 bars	298.2/313.2 K
	1, 15 wt % [mTBNH][OAc]	40 bars	298.2/313.2 K

The IL concentration in the permeate and retentate solution was determined at 303.2 K using an Anton Paar Abbemat 5010 refractometer, with an uncertainty of 2 × 10^–5^ nD. A calibration curve was previously performed using standards with different compositions (uncertainty of 10^–4^ g).

IL rejection was determined by eq 2:
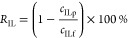
2where *R*_IL_ is the rejection of IL, *c*_ILp_ is the concentration of IL in the permeate solution, and *c*_ILf_ is the concentration of IL in the feed solution.

### 2.3 Membrane Cleaning

The process of membrane fouling results in the loss of performance (in terms of flow) of a membrane because of the presence of suspended or dissolved substances in the membrane’s pores.^29^ In order to avoid such process, chemical cleaning of the membrane was carried out between each experiment. In this way, guaranteeing the same membrane performance throughout the tests was possible.

The cleaning procedure consisted of first permeating the membrane with an aqueous solution of sodium hydroxide (0.1%) for 45 min (at 313.2 K and 15 bars), then permeating the membrane with an aqueous solution of sulfuric acid (0.2%) for 45 min (at 313.2 K and 15 bars) and finally check the permeation flux of the membrane with ultrapure water at 298.2 K and 10 bars (Figure S6). If the flow is lower than that obtained in the previous test, the cleaning process was repeated. After use, all membranes were rinsed with water and stored in an aqueous solution of 1% sodium metabisulfite to prevent bacterial growth.

## 3 Results

Membrane filtration is a process of removing/separating substances by forcing the solution to permeate through a porous medium. Different factors can affect membrane efficiency. The main membrane characteristics controlling the filtration efficiency are the membrane properties, pore size, hydrophobicity, and pore size distribution and material. On the other hand, the solution properties, solution concentration, particle size, and nature of compounds are also essential.^27^ Considering this, a study was conducted with various types of membranes with different pore sizes to evaluate the recovery of superbase IL from an aqueous solution.

### 3.1 Membrane Screening

The volumetric flux and rejection of substances can be affected by several filtration factors, such as transmembrane pressure, temperature, osmotic pressure, substance concentration, and other membrane characteristics (porosity, density). At first, eight membranes were selected (MP005-MF, PT-UF, GH-UF, DL-NF, TS80-NF, NF270-NF, BW30LE-NF, and UTC-73A-RO) with different porosities and densities. The permeation flux and the IL rejection of diluted IL aqueous solutions (1 wt %) were determined by applying a transmembrane pressure of 10 bars, at 298.2 K, on these membranes. The stability and integrity of the IL after filtration were investigated by NMR and FTIR. The band assignments were performed according to the IR spectrum table by the frequency range reported in the literature.^30^ Characteristic IL absorption bands related to C–N stretching (1342–1266 cm^–1^), O–H bending (1440–1395 cm^–1^), C=N stretching (1690–1640 cm^–1^), and O–H stretching (3200–2700 cm^–1^) were observed in all spectra, suggesting no changes in the IL structure after membrane treatments (Figure S4). The ^1^H-NMR and ^13^C-NMR spectra with chemical shifts of ILs before and after permeation are presented in Figures S2 and S3. The chemical shift difference of the peak’s signals of both IL before and after permeation was insignificant. For example, the chemical shift of hydrogens in the acetate of [mTBDH][OAc] presented a value of 1.60 ppm for both samples before and after permeation. Therefore, the IL remains intact without modification in the chemical structure or molar ratio of IL cations/anions, remaining stable and intact after permeation.

**Figure 1:**
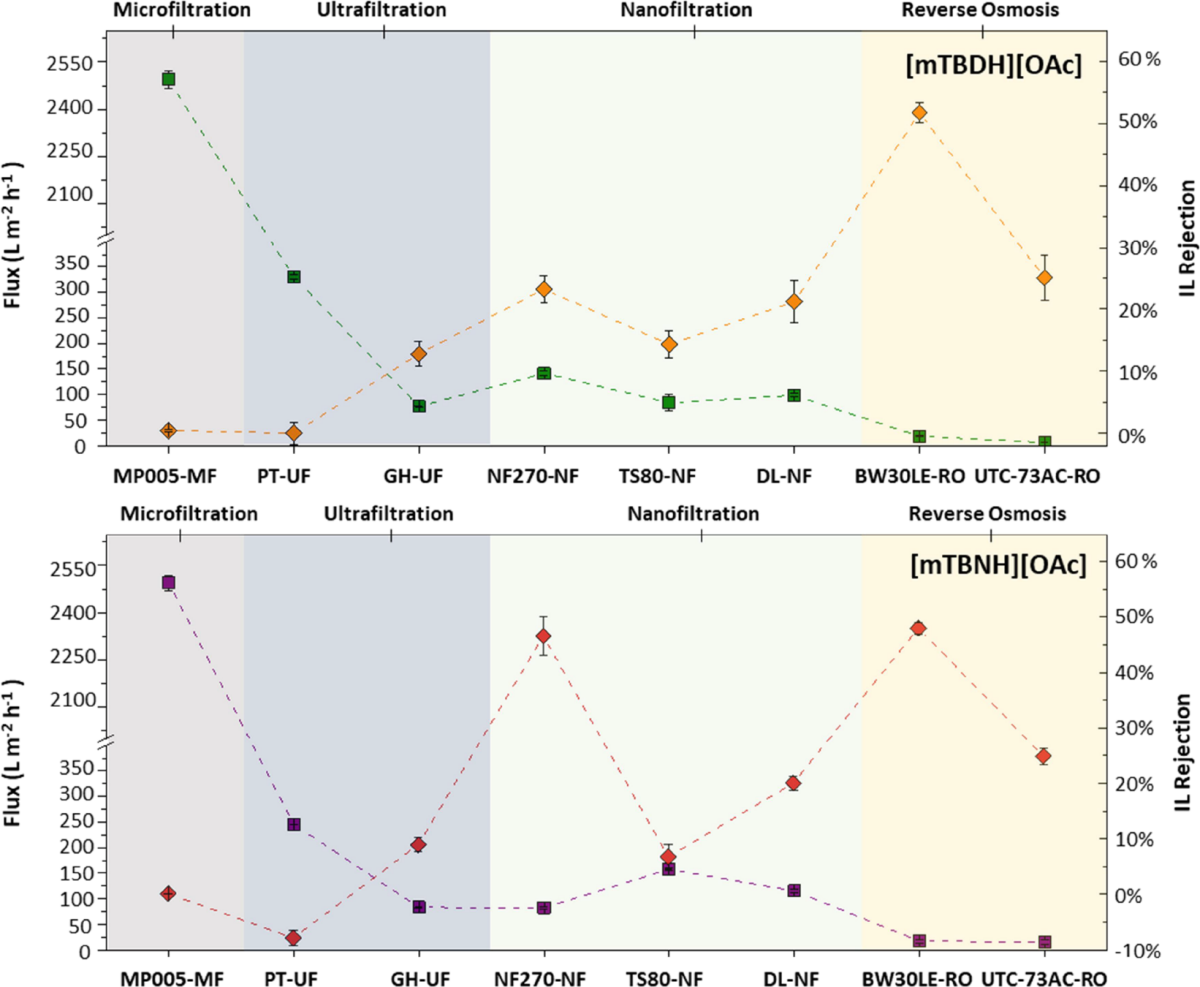
Effect of membrane on the volumetric flux ((green square) [mTBDH][OAc] and (purple square) [mTBNH][OAc]) and IL rejection ((orange diamond) [mTBDH][OAc] and (red diamond) [mTBNH][OAc]). Conditions: solution of 1 wt % of [mTBDH][OAc] or [mTBNH][OAc], 10 bars, 200 rpm at 298.2 K. Dashed lines are visual guides.

Figure 1 shows the experimental volumetric flux and IL rejection of aqueous solutions containing 1 wt % of IL. It can be seen that the volumetric fluxes, at 10 bars, followed the order of MP005-MF > PT-UF > TS80-NF > DL-NF > GH-UF > NF270-NF > BW30LE-NF > UTC-73A-RO. The correlation between membrane porosity and volumetric flux is presented in Figure S7. The maximum volumetric flux was observed for the solution of 1 wt % of [mTBNH][OAc] with the membrane MP005-MF (2494.8 L m^–2^ h^–1^) and the minimum for the solution of 1 wt % of [mTBDH][OAc] with the UTC-73A-RO membrane (5.8 L m^–2^ h^–1^). The differences in membrane porosity may explain this behavior.

Microfiltration membranes (MF) have pores of up to 0.1 μm, which do not offer any resistance to the passage of IL molecules. Furthermore, they are commonly used at pressures below 1 bar, so at pressures of 10 bar, flows tend to be higher with almost no IL rejection.^31^ Ultrafiltration membranes (UF) have a smaller pore size, in the nanometer range (2–100 nm), in addition to higher porosity, which leads to a certain resistance to the passage of the IL.^32^ Nanofiltration (NF) membranes, on the other hand, have a pore size of less than 1 nm and are able to retain part of the IL.^33^ In the case of the NF270-NF membrane, rejection values of 46.5% were obtained. Unlike others, RO membranes are not porous but dense, and this causes the IL to diffuse through the membrane.^34^ Because of its larger molecular volume, IL tends to have a slower diffusion through the membrane when compared to water molecules, and therefore, the rejection tends to be higher for this type of membrane (>45%). In the case of [mTBNH][OAc], the IL rejection order is BW30LE-NF > NF270-NF > UTC-73A-RO > DL-NF > TS80-NF ≈ GH-UF > MP005-MF > PT-UF.

However, the size of the molecules is not the only factor that affects the separation efficiency, the interactions of the molecules with the membrane as well as the charge of the ions or molecules influence the retention.^35^ Because the ion charge exclusion depends on the membrane charges, the ionic force, and the ion valence.^36^ Avram et al.^20^ observed that the size-based separation alone was ineffective in separating IL and low molecular weight sugars (glucose). The authors indicate that controlling the thickness and structure of the layer was essential to maximize the rejection of sugar. In addition, the volumetric flux is reduced, and RO generally requires high transmembrane pressures to operate in industrial processes.

Except for the PT-UF membrane, part of the IL can be retained in all other membranes, whereas the water permeates. In the case of PT-UF, most water can be retained, whereas the IL permeates through the membrane. In this case, the limiting factor of the separation was the affinity between the IL molecule and the membrane surface and not the porosity/density of the membrane.

Remarkably, the NF270-NF membrane showed IL rejection rates comparable to RO membranes (BW30LE-RO and UTC-73 AC-RO). This implies that for some situations, it is possible to obtain the same IL rejection but with a much higher volumetric flow (4.5 times), allowing for a more efficient filtration process from an operational point of view.

Based on the membrane screening results, the NF270-NF (IL rejection >23%) and BW30LE-RO (IL rejection >48%) membranes were selected, and the effect of pressure, feed IL concentration, and the effect of temperature were further evaluated.

### 3.2 Pressure Effect

**Figure 2:**
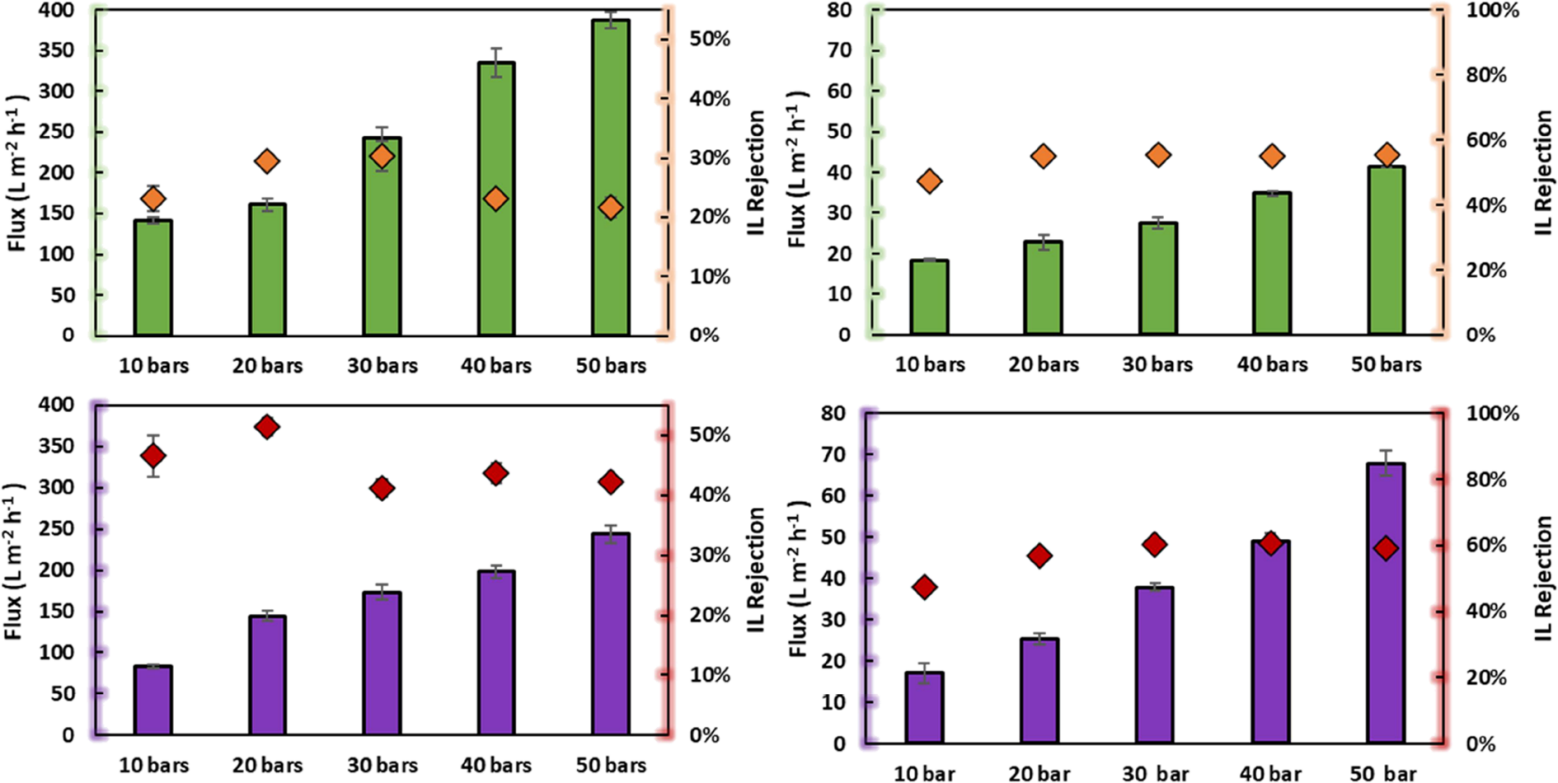
Effect of pressure on the performance of NF270-NF (left) and BE30LE-RO (right): volumetric flux (green square) [mTBDH][OAc] and (purple square) [mTBNH][OAc] and IL rejection (orange diamond) [mTBDH][OAc] (red diamond) [mTBNH][OAc]. Conditions: 1 wt % [mTBDH][OAc] or [mTBNH][OAc], 10, 20, 30, 40, 50 bars, 200 rpm at 298.2 K.

In order to evaluate the pressure effect during the filtration of superbase ILs with NF270-NF and BW30LE-RO membranes, a series of filtrations, presented in Figure 2, were performed at different pressures (10, 20, 30, 40, and 50 bars). During the filtration of an IL solution ([mTBDH][OAc] and [mTBNH][OAc])) with the NF270 membrane, the IL rejection increases with increasing pressure, reaching a maximum value followed by a decrease. This behavior differs from most trends reported in the literature for nanofiltrations of binary mixtures. At first, an increase in retention with increasing pressure is observed, followed by a smaller increase or stabilization at higher pressures.^37−39^ For example, Wang et al.^25^ evaluated the filtration behavior of ILs ([AMIM]Cl, [BMIM]Cl, and [BMIM][BF_4_]) in an aqueous solution by NF270-NF. The authors observed that the permeate flux and IL rejection increased with the pressure applied at a constant IL concentration.

However, a maximum retention peak with increasing pressure for some systems has been reported.^40,41^ The authors attribute this behavior to the effect of an increase in the polarization layer with pressure when the cross-flow velocity is low.^41^ Nevertheless, in the results obtained in this study, the volumetric flux of the permeate tends to increase linearly with pressure. Xu and Lebrun^42^ consider this linearity because of the absence of concentration polarization. Therefore, the decrease in rejection after a given pressure cannot be justified regarding the concentration polarization phenomenon.

Because NF270-NF is a porous membrane, the IL would be expected to enter the membrane pore (whose cut-off diameter is 200–400 Da) and remain partially retained due to membrane surface forces.^43^ As pressure increases, surface forces remain constant while drag forces increase because of increased pore flow. At low pressures, surface forces tend to be stronger than drag forces. Therefore, the IL flow remains low, while the water flow increases with pressure, resulting in an increased IL rejection. Above a certain pressure, drag forces become higher than surface forces, and, consequently, the solute transfer increases and the retention decreases.^44^

Abels et al.^26^ evaluated the IL rejection of IL/water mixture with IL mass fraction ranging from 0 to 80 wt % by two commercially available polyamide and one polyimide membranes (Desal DL, Desal DK, and Starmem 240). At low IL concentrations, the effect of pressure played a significant role in the IL rejection. However, at high IL concentrations, the pressure effect is less pronounced.

For the BW30LE-RO membrane, increased retention is observed with increasing pressure, followed by stabilization at higher pressures. Concerning volumetric flow, the increase in pressure results in a linear increase in volumetric flow. This plateau can be beneficial in the industrial operation of RO membranes because at higher pressures, the IL rejection rates are the same as those at lower pressures. Still, the permeate fluxes are higher, making the process more efficient.^45^

### 3.3 IL Feed Concentration Effect

In general, membrane permeation is more difficult for large molecules than for smaller molecules, so the transmembrane pressure tends to increase with the size of the molecule. However, the composition of the medium, more specifically the concentration, also has an effect on membrane performance.^46^

**Figure 3:**
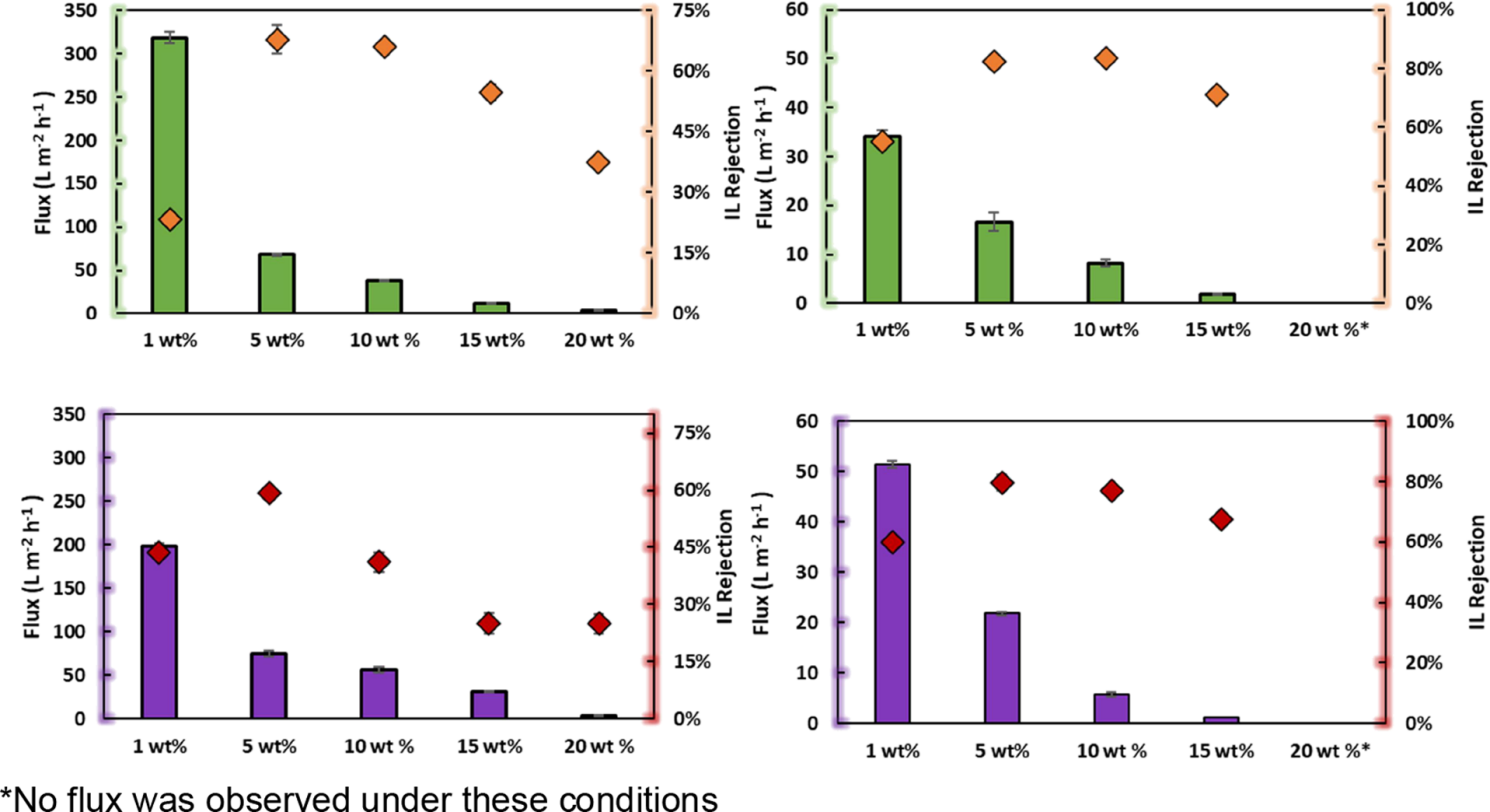
Effect of feed IL concentration on the performance of NF270-NF (left) and BW30LE-RO (right): volumetric flux (green square) [mTBDH][OAc] and (purple square) [mTBNH][OAc], and IL rejection (orange diamond) [mTBDH][OAc] (red diamond) [mTBNH][OAc]. Conditions: 1, 5, 10, 15, and 20 wt % [mTBDH][OAc] or [mTBNH][OAc] 200 rpm at 40 bars and 298.2 K. *No flux was observed under these conditions.

The relation between membrane performance (volume flow and IL rejection) and IL concentration in the feed solution is shown in Figure 3. With the increased IL concentration in the feed solution, a reduction of the volumetric flux is observed. For example, for the BW30LE-RO membrane, no volumetric flux was observed for the concentration of 20 wt % of IL.

Regarding IL rejection, the results show that the increase in concentration decreases IL retention. Wang et al.^25^ reported the same behavior with the filtration of aqueous solutions of [BMIM][Cl] and [AMIM][Cl] by nanofiltration membranes (NF90 and NF270). This behavior is characteristic of this membrane type and is generally interpreted by the shielding phenomenon.^38,47^ This effect is mainly attributed to the cation shielding of the effective charge of the membrane. This characteristic can be explained by the electrical repulsion becoming less efficient at higher concentrations because there is a tendency to form an IL film on the membrane that gradually neutralizes the charges on its surface. Consequently, the repulsive forces decreased, so the rejection rate was slightly reduced.^48^

This effect tends to be weak at low concentrations, so high retention is expected. However, a low IL retention rate was observed for the 1 wt % solution. This behavior is related to the high volumetric flux, in which the drag forces overcome the surface forces, decreasing IL rejection.^44^ When the concentration is higher, this effect tends to be more prominent, and the membrane potential weakens. Abels et al.^26^ observed at higher IL concentrations that no separation of IL from the mixture was achieved using polyamide membranes (Desal DL and Desal DK). Furthermore, as the repulsion between the membrane and the ions decreases, they tend to cross the membrane more quickly, thus dragging the other ions and retention is thus decreased.^44^

Therefore, the concentration of the IL solution plays a crucial role in the case of membrane fouling, which alters the performance characteristics, resulting not only in a significant decrease in flux or permeability but also in reduced IL rejection.^47^

### 3.4 Temperature Effect

The nanofiltration and RO membranes are designed to operate at room temperature. However, their use at temperatures above ambient may provide better performance depending on the conditions and feed solution.^49^

**Figure 4:**
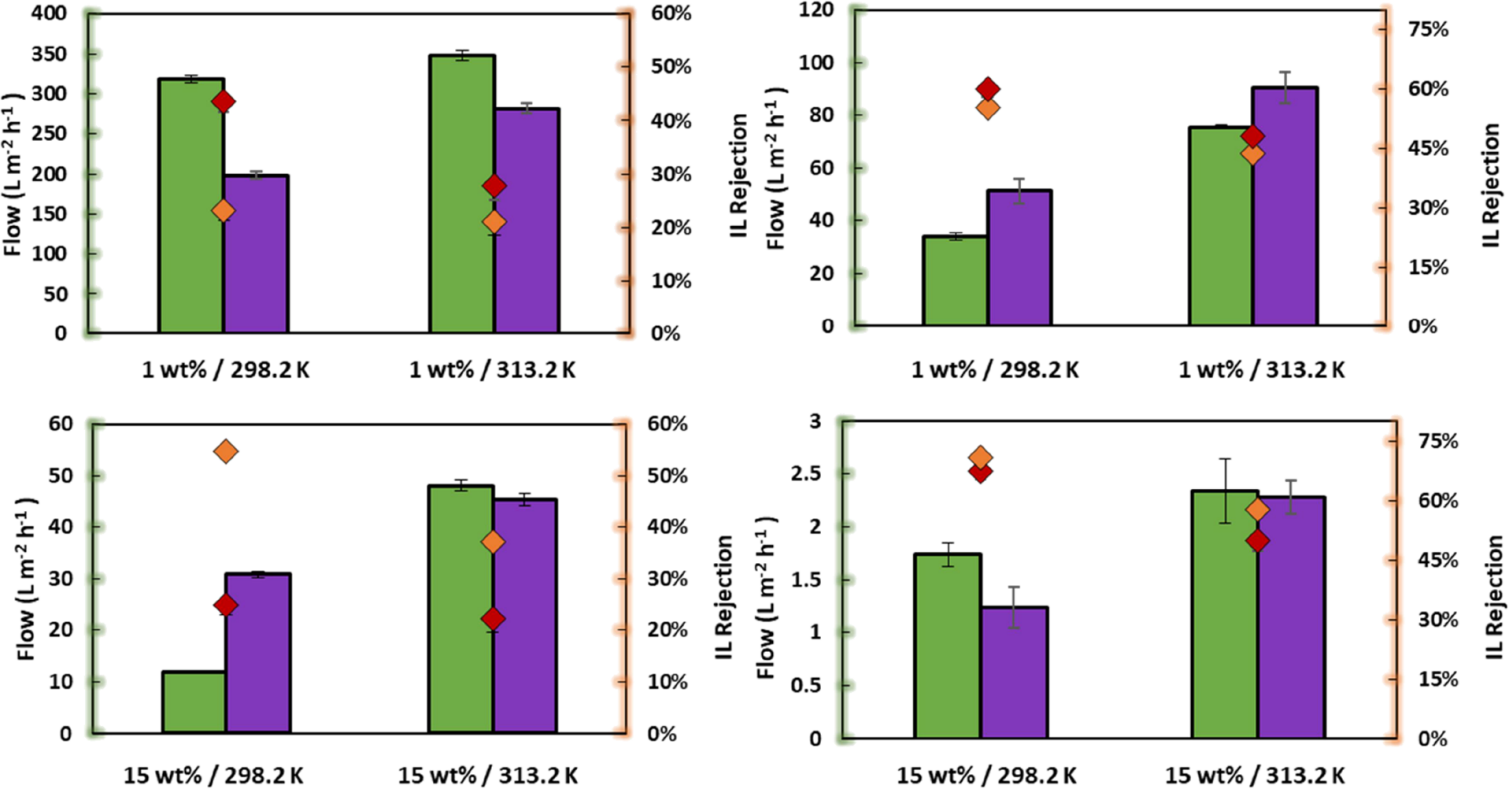
Temperature effect on the performance of NF270-NF (left) and BW30LE-RO (right): volumetric flux (green square) [mTBDH][OAc] and (purple square) [mTBNH][OAc], and IL rejection (orange diamond) [mTBDH][OAc] (red diamond) [mTBNH][OAc]. Conditions: 1 and 15 wt % [mTBDH][OAc] or [mTBNH][OAc] 200 rpm at 40 bars and 298.2 or 313.2 K.

The effect of temperature (studied at 298.2 and 313.2 K) on membrane performance is shown in Figure 4. It is shown that while the volumetric fluxes of the membrane improve, the rejection rate decreases with increasing temperature. The temperature increase in solute transport is mainly related to the cumulative effect of reducing solvent viscosity and increasing ion diffusivity. Therefore, this effect is more pronounced for more concentrated solutions (15 wt %).^48^ Nilsson et al.^50^ did not observe significant changes in the isoelectric point of the NFT-50 membrane (Alpha Laval) with temperature variation, concluding that the membrane charge properties are not significantly affected by the temperature increase. However, other parameters such as solvent viscosity, solute diffusivity, and structural parameters tend to be affected with increasing temperature. The effect of modifying these parameters with temperature has a direct impact on the passage of ions.^51^ However, Abels et al.^26^ observed that the increase in temperature had a minor effect on the permeability of IL, even though the viscosity decreased. In general, it is not enough to consider only the change in solvent viscosity or solute diffusivity to explain the increase in volumetric flux and the reduction in IL rejection. A study of the structural parameters of the membrane, which were not studied in this work, is necessary.^51^

Therefore, for the conditions tested, increasing temperature results in an improvement in volumetric membrane fluxes and a slight reduction in IL retention. The use of temperature can be a solution for high concentrated or viscous solutions.

### 3.5 Multicycle Filtration

Multicycle membrane filtration experiments were used to simulate the continuous operation membrane that is foreseeable at an industrial scale. The cycles using the nanofiltration and the reverse osmose filtration membranes were performed. Each nanofiltration cycle included 15 min of filtration followed by a chemical and water cleaning process. The RO filtration cycles comprised 90 min of filtration followed by a chemical and water cleaning cycle. As shown before, a long time was required because of the low volumetric flux obtained with the RO membrane.

**Figure 5:**
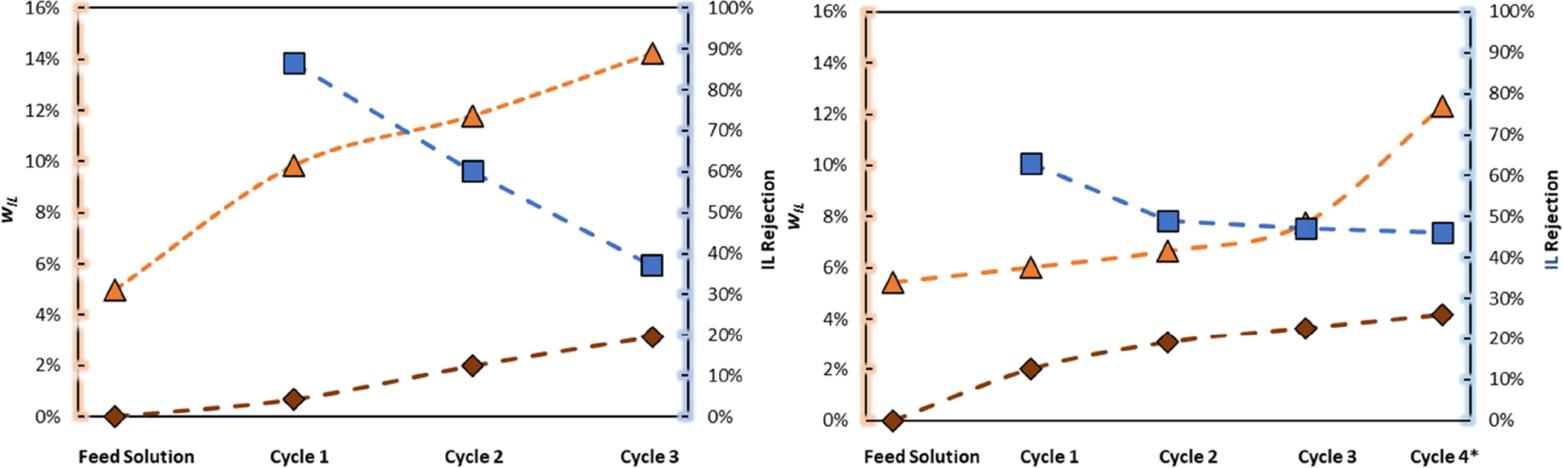
Multicycle membrane filtration experiments of NF270-NF (left) and BW30LE-RO (right): [mTBNH][OAc] concentration in the retentate (triangle) [mTBNH][OAc] concentration in the permeate (diamond) [mTBNH][OAc], and IL rejection (square) of each cycle. Conditions: 5 wt % [mTBNH][OAc] 200 rpm at 40 bars and 298.2 K. Dashed lines are visual guides.

At each cycle, an increase in the concentration of the retentate was observed. As previously discussed, this behavior directly impacts the volumetric flux and retention of the IL because of the increase in the concentration of the solution (Figure 5). For the NF270-NF membrane, the permeate concentration increases with each cycle, and this means that the IL retention efficiency decreases with the increase in solution concentration.

During the cycles, it was possible to concentrate the initial solution (5 wt %) of IL about 1.5 and 2.9 times for NF270-NR and BW30LE-RO, respectively. In the nanofiltration, cycle 4 is the longest cycle (approx 50 min), and in this cycle, it was possible to concentrate an initial solution from 7.7 to 12.3 wt % of [mTBNH][OAc]. This longer cycle allowed us to observe that there is a tendency to stabilize the IL retention as well as the concentration in the permeate with increasing filtration time. Another critical point to highlight is that the concentration of IL in the permeate of NF270-NF is higher than the concentration of IL in the BW30LE-RO permeate.

**Figure 6:**
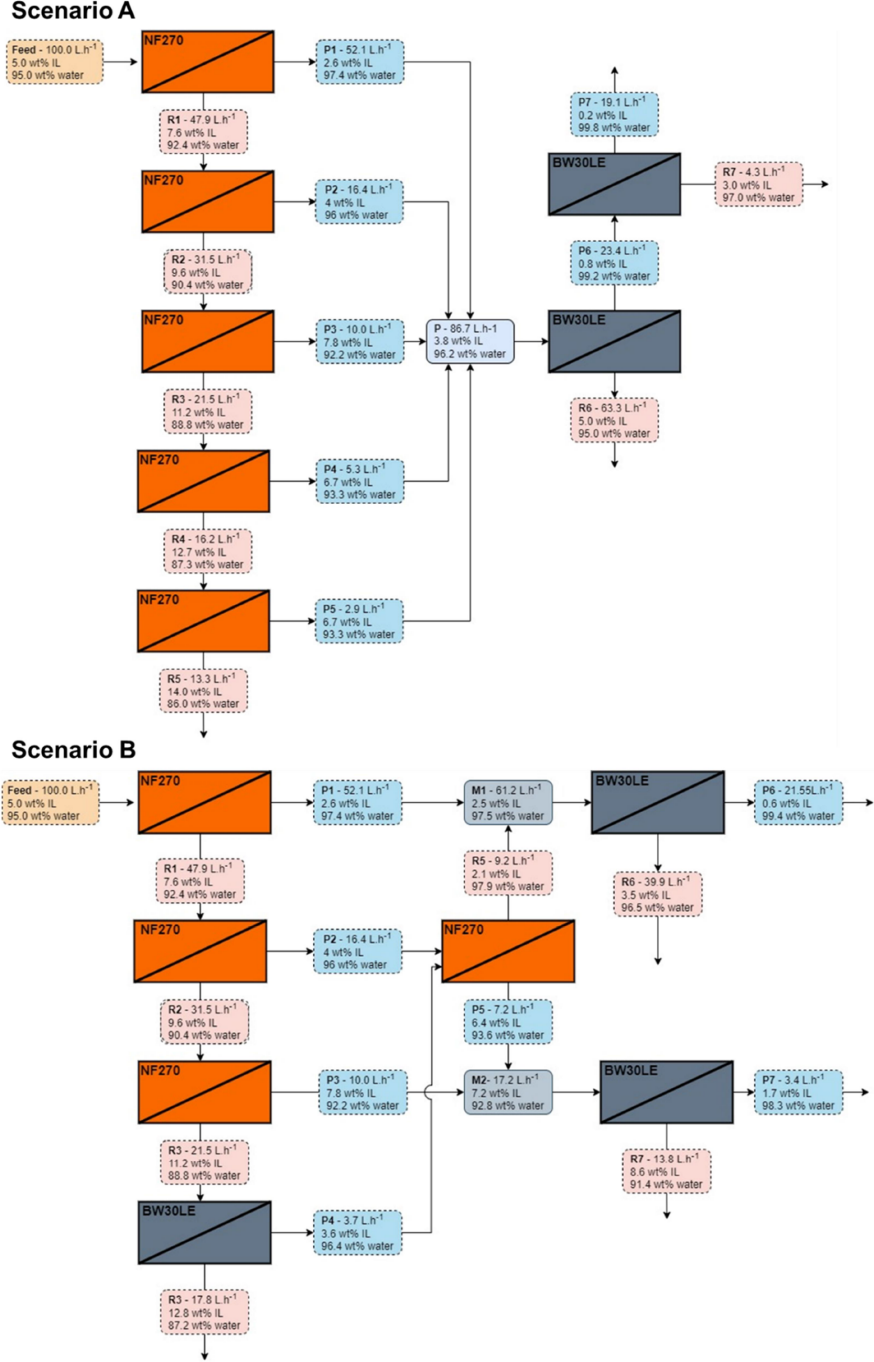
Flowchart of the proposed filtration Scenario A and Scenario B combining nanofiltration and RO membrane. Conditions: 5 wt % [mTBNH][OAc] 200 rpm at 40 bars and 298.2 K.

With the parameters of IL rejection, permeate, and retentate concentration, as well as the volumetric fluxes, it was possible to propose filtration scenarios combining nanofiltration membrane and RO (Figure 6). Calculations were performed for filtration of a solution containing 5 wt % of [mTBNH][OAc] and flow of 100 L h^–1^ in a series of NF270-NF and BW30LE-RO membranes. In scenarios A and B, the IL permeate concentration increases, and the permeate flux decreases every new cycle. This behavior is a result of the increase in the IL feed concentration in each new cycle, which as verified, affects the IL rejection. At the end of the fifth cycle in scenario A, it was possible to concentrate the IL in a solution containing 14 wt % (R5). The permeate streams were combined (mixture containing ≈3.8 wt % IL) and filtered through two BW30LE-RO membranes. From the BW30LE membranes, two streams resulted, the permeate stream (P7) with a diluted IL solution (0.2 wt % IL) and the retentate stream (R6) with a concentration of 5 wt % IL, the same concentration as the feed. Therefore, the retentate stream can be fed back into the system, as shown in the diagram (Figure 6).

In scenario B, the permeate stream from the second nanofiltration membrane (P2) was filtered by the NF270-NF membrane. The retentate from that filtration (R5) was mixed with the permeate stream from the first membrane (P1) and fed into a BW30LE-RO. Then, the P5 stream was combined with the permeate of the third NF270-NF (P3) and fed into a RO membrane. Thus, it was possible to obtain a concentrated IL stream (R3 ≈ 12.8 wt % IL). The stream R7 (≈8.6 wt % IL) can be fed back into the third NF270-NF membrane with stream R2, the P4 stream can be combined with P2 stream and be fed into the NF270-NF, and streams R6 and P4 (≈3.5 wt % IL) mixed and fed through a RO membrane.

In general, under the optimal experimental parameters, the aqueous solution of IL at 5 wt % can be concentrated to approximately 14 wt %. The configuration of the membranes in the proposed scenario allowed to obtain a water stream practically without IL (≈0.2 wt % IL), an advantage for the process because the main purpose is to remove water from the IL solution.

Furthermore, the streams with low IL concentration can be fed into other membranes, for example, RO membranes, which would allow a significant reduction in IL loss, resulting in a stream with pure water and a concentrated stream in IL. Combining nanofiltration and RO membranes is essential to avoid IL loss during filtration and ensure minimal flow to feed other membranes. The results showed that the nanofiltration membrane (NF270-NF) has IL rejection rates similar to RO (BW30LE-RO) for dilute solutions, but with higher fluxes. These results provide the fundamental data necessary for applying nanofiltration and RO technology to preconcentrate IL from spinning bath.

It is important to emphasize that the concentration reached at the end of the filtration (≈14 wt %) is far from the concentration required (≈80 wt %) to reuse IL with the same cellulose dissolution capacity. Elsayed et al.^9^ used a set of heat treatment operations (centrifugal evaporator) to concentrate the spinning bath. With this approach, obtaining an IL solution with low residual water content (2.2–3.1 wt %) was possible. However, the authors reported that the energy demand for the recovery of dilute IL solutions (0.1–1.5 wt % IL) is tremendously high. Therefore, it reinforces this study approach to utilize membrane treatment to preconcentrate the spinning bath solution.

In order to compare the IL recovery technique by membrane filtration and distillation, energy expenses were preliminarily calculated to support the proposed conclusions. For this, the software Aspen Plus V12.1 was used. The model design was based on a COSMO-SAC method. Distillation was simulated with a Radfrac distillation column with a reboiler only (Figure S8). The feed stream was a solution containing 5 wt % of [mTBNH][OAc] and a flow of 100 L h^–1^. It was defined as a specification that the IL current should have a concentration of 15 wt % IL, the same value obtained for the membrane scenarios. To determine the energy expenses of the membranes, a hydraulic turbine was used, and the pressure set was 40 bars.

For distillation, an energy expense of 4790.6 W/kg_Feed_ was obtained, while for filtration, the expenditure was only 2.6 W/kg_Feed_. The energy expenditure required for the membranes is lower when compared to distillation because it is not necessary to vaporize water, which, in this scenario, comprises up about 95% of the solution. These results emphasize the idea that membrane filtration technology should be considered in the preconcentration stage of the IL spinning bath because there is a large amount of water to be removed at this stage.

On the other hand, distillation can be applied to a subsequent concentration step when the solution is already more concentrated, and the IL rejections by the membranes tend to decrease.

## 4 Conclusions

In this work, the filtration behavior of aqueous solutions of two superbase ILs, [mTBDH][OAc] and [mTBNH][OAc], using microfiltration, ultrafiltration, nanofiltration, and RO membranes were studied. Nanofiltration (NF270-NF) and RO (BW30LE-RO) membranes showed the highest capacity for retention/separation of IL from the diluted aqueous solution (>45%). For these membranes, the volumetric flux of permeate increased linearly with increasing pressure applied at constant IL concentration and decreased with increasing IL concentration in the feed solution at constant pressure.

Compared to the nanofiltration membrane (NF270-NF), the reverse osmosis membrane (BW30LE-RO) showed the highest retention because of its smaller pore size. Regarding IL rejection, the results show that the increase in concentration decreases IL retention. The use of higher temperatures (313.2 K) resulted in an increase in the volumetric flow of the membrane and consequently a reduction in the IL rejection rate. Using the filtration membrane in a series of cycles allowed to concentrate the initial solution of 5 to 14 wt % of IL.

From these results, it is possible to remark that membrane filtration can hardly be used as a single step for separating IL from water because the maximum IL concentration obtained (14 wt %) is lower than the desired (80 wt %) for IL reuse. Therefore, complete separation of IL and water can be achieved by combining different separation methods, such as distillation and membrane separation. Thus, it is possible to reach the desired IL concentration using distillation and reduce the energy demand for diluted solutions with membrane filtration.

## Supporting Information Available

IL characterization (^1^H-NMR); specification of membranes; water permeation flux (NF270-NF and BW30LE-RO); and retentate and permeated characterization (^1^H-NMR) (PDF)
