# Supplementary material for: Preconcentration of Superbase Ionic Liquid from Aqueous Solution by Membrane Filtration
Source: Ind Eng Chem Res. 2022 Sep 23;61(39):14626–36. doi: 10.1021/acs.iecr.2c02217 (PMC9777811; doi:10.1021/acs.iecr.2c02217)
Supplement: Supplementary file 1 — ie2c02217_si_001.pdf [file ie2c02217_si_001.pdf]

Supplementary Material

Preconcentration of super-base ionic liquid from aqueous solution by membrane filtration

Filipe H. B. Sosa[1], Pedro J. Carvalho[1], and João A.P. Coutinho[1*].

[1]CICECO – Aveiro Institute of Materials, Department of Chemistry, University of Aveiro, 3810-193 Aveiro (Portugal)

*Corresponding author: João A. P. Coutinho, E-mail address: jcoutinho@ua.pt

Number of figures: 8

Number of tables: 1

S1 – Ionocell process

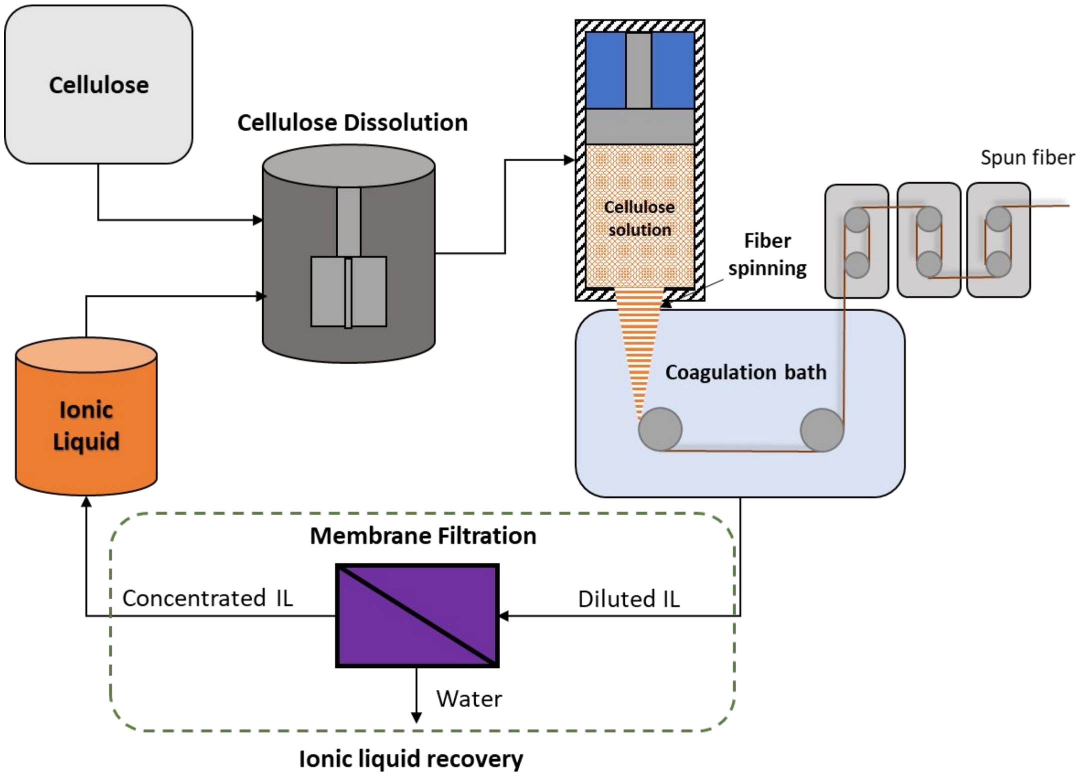

Figure S1. Diagram of the Ioncell process revealing dissolution, fiber spinning, and ionic liquid recovery steps.

S2 – Ionic liquid characterization

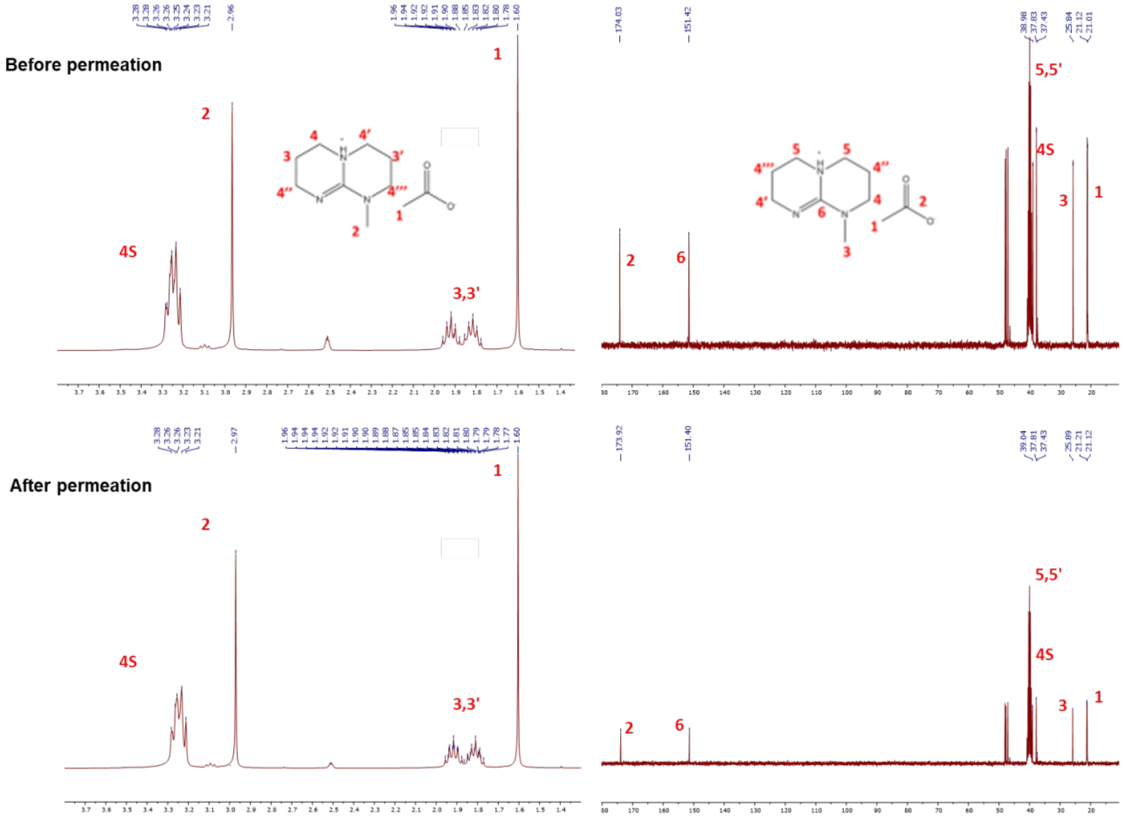

Figure S2. ^{1}H-NMR and ^{13}C-NMR spectra of [mTBDH][OAc] (top) and permeated aqueous [mTBDH][OAc] solution (bottom).

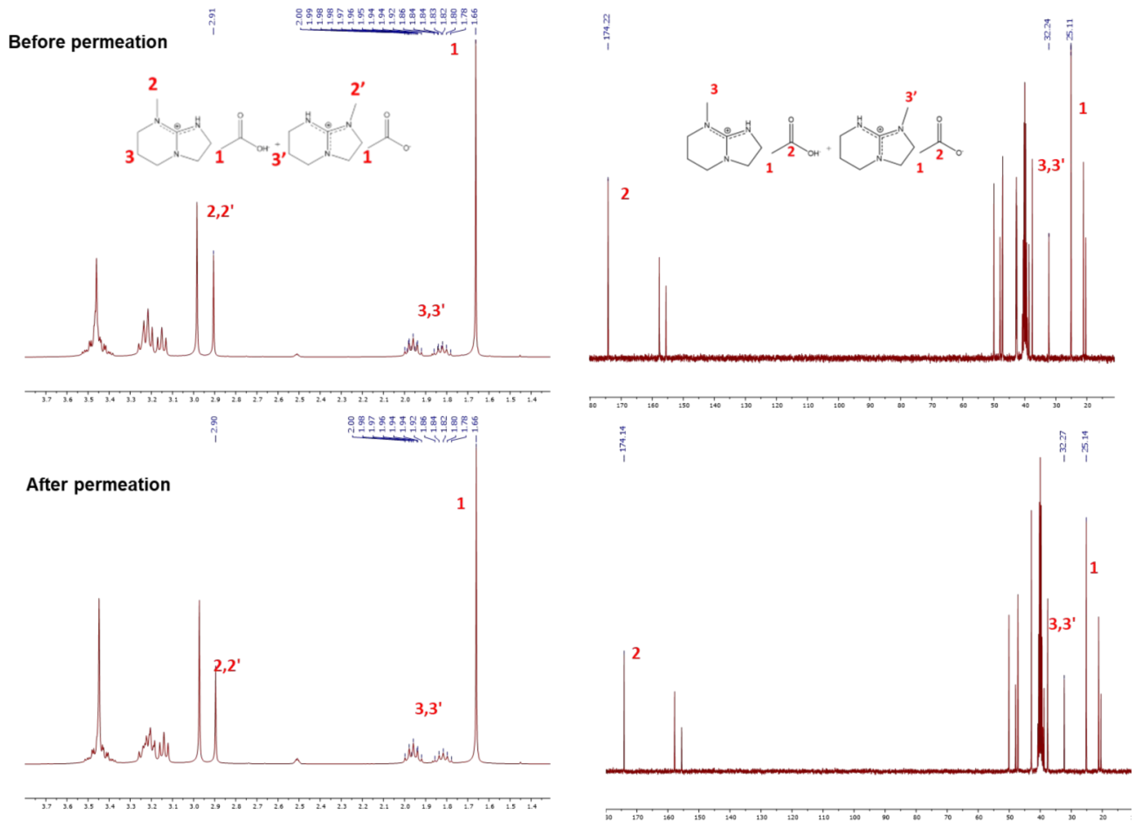

Figure S3. ^{1}H-NMR and ^{13}C-NMR spectra of [mTBNH][OAc] (top) and permeated aqueous [mTBDH][OAc] solution (bottom).

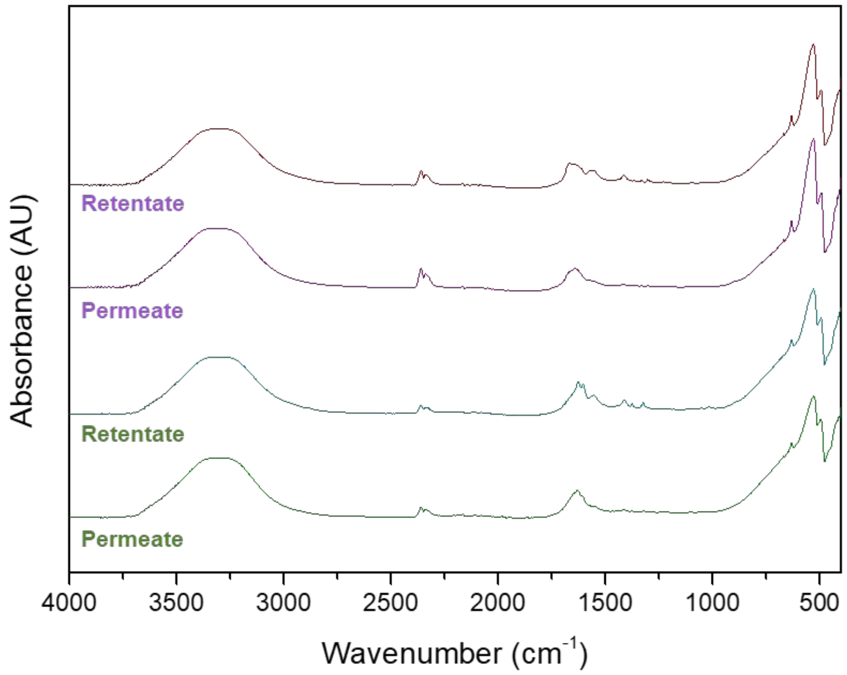

Figure S4. FTIR−ATR spectra of solution after and before membrane permeation of [mTBDH][OAc] (—) or [mTBNH][OAc] (—) Conditions: Feed solution = 15 wt% [mTBDH][OAc] or [mTBNH][OAc], 40 bars, 200 rpm at 298.2 K with BW30LE-RO.

S3 - Supplementary experiments

The selection of feed volume (75 ml) is related to the influence of the variation of the concentration solution inside the cell filtration over time. Since the dead-end filtration cell works in batch mode, as the solution is permeated, the concentration of IL inside the cell tends to increase. This increase in IL concentration within the filtration cell directly affects the IL permeation and rejection fluxes. Thus, several tests were carried out with different masses of initial solution (Figure S2) and it was found that for masses greater than 74 g the influence on the permeation flow curve is minimal, so we can guarantee that this influence on the tests will be minimized.

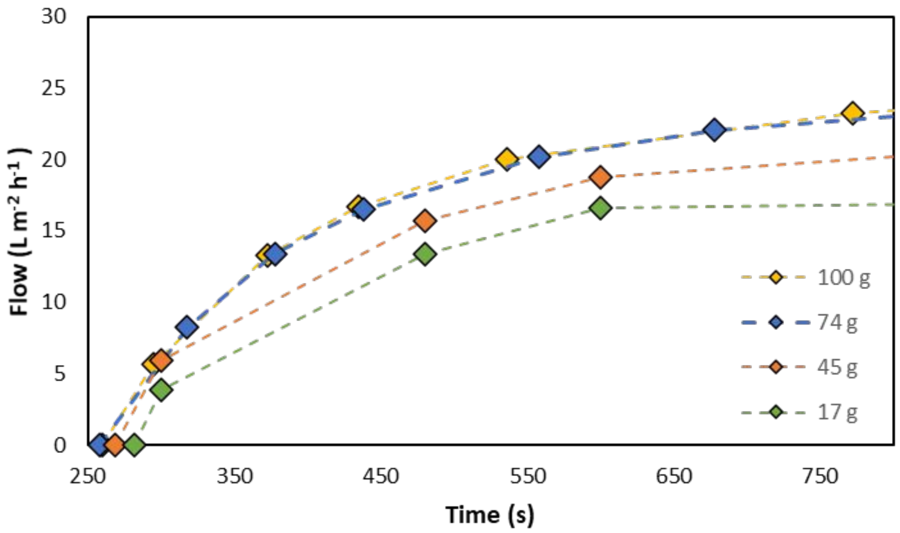

Figure S5. Mass feed influence in the permeation flux of the BW30LE-RO membrane at 298.2 K and 10 bars. Conditions: 5 wt% [mTBNH][OAc], 40 bars, 200 rpm at 298.2 K.

S4 - Specification of membranes

Table S1. Specifications of membranes.

Manufacturer	Membrane	Material	Molecular weight cut-off (Da)*	pH
Microdyn Nadir	MP005	Polysulfone (PES)	0.05 μm	0 – 14
Suez (GE)	GH	Polyamide-TFC	2000 Da	1 – 11
Suez (GE)	PT	Polyethersulfone	5000 Da	1 – 11
FilmTec	NF270	Polyamide – TFC	~ 200 – 400	2 – 11
TripSep	TS80	Polyamide – TFC	~ 150	2 – 11
Suez (GE)	DL	Polyamide – TFC	~ 150 – 300	2 – 10
Dow Filmtec	BW30LE	Polyamide – TFC	N/A	2 – 11
Toray	UTC-73AC	Polyamide – TFC	N/A	2 – 11

*Reported by the supplier

S5 – Water permeation flux

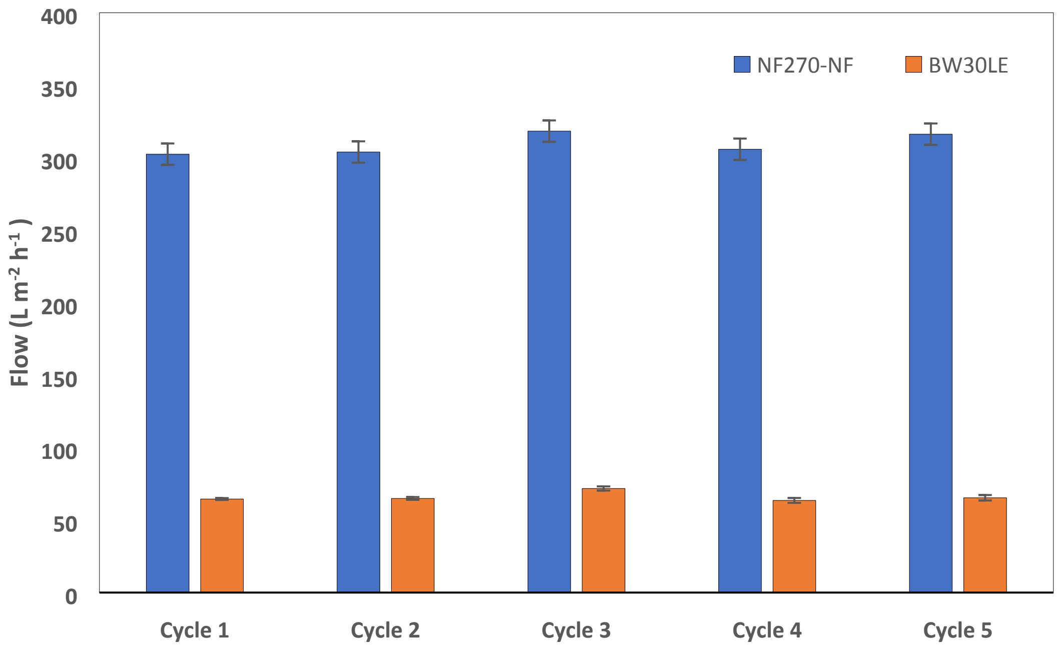

Figure S6. Water permeation flux of the membrane at 298.2 K and 10 bars after cleaning.

S6 – Correlation between membrane porosity and volumetric flux

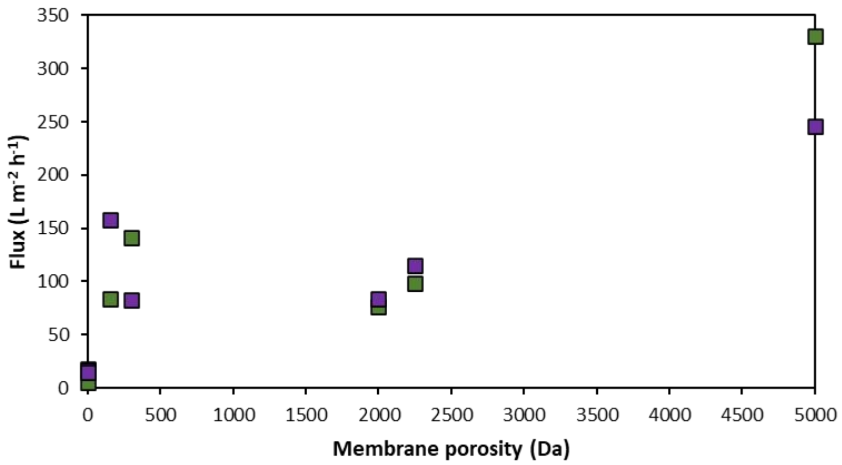

Figure S7. The correlation between membrane porosity and volumetric flux [mTBDH][OAc] (■) and [mTBDH][OAc] (■). Conditions: solution of 1 wt% of [mTBDH][OAc] or [mTBNH][OAc], 10 bars, 200 rpm at 298.2 K.

S7 – Energy expenses

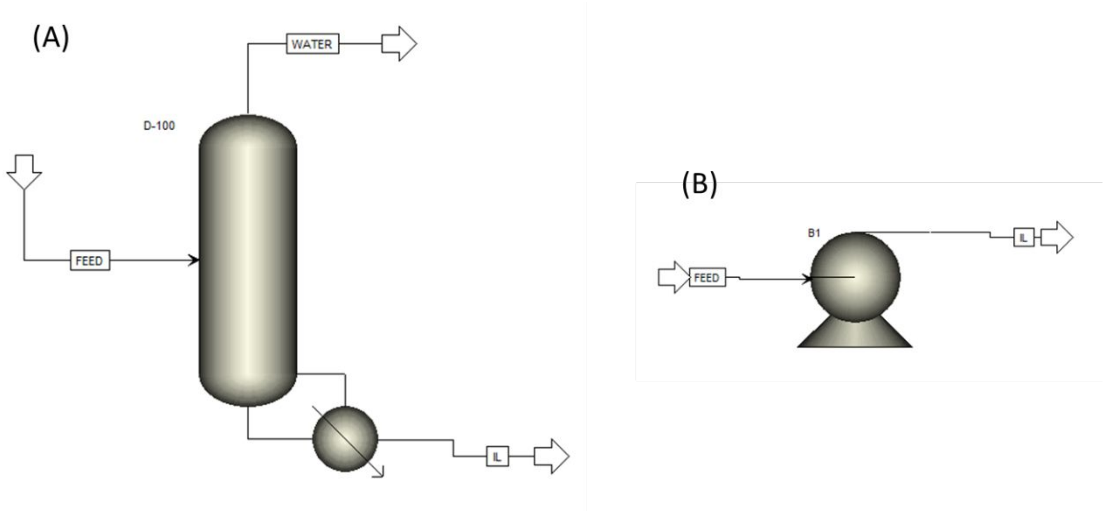

Figure S8. Flowchart of the equipment used to simulate the energy expenditure of (A) distillation and (B) membrane filtration of a solution containing 5 wt% of [mTBNH][OAc] and flow of 100 L h^{-1}.
